# Exosomes in the pathogenesis and treatment of cancer-related cachexia

**DOI:** 10.1186/s12967-024-05201-y

**Published:** 2024-04-30

**Authors:** Qin Ru, Lin Chen, Guodong Xu, Yuxiang Wu

**Affiliations:** Institute of Intelligent Sport and Proactive Health，Department of Health and Physical Education, Jianghan University, Wuhan, 430056 China

**Keywords:** Cancer-related cachexia, Exosomes, Non-coding RNAs, Muscle atrophy, Lipidolysis

## Abstract

Cancer-related cachexia is a metabolic syndrome characterized by weight loss, adipose tissue decomposition, and progressive skeletal muscle atrophy. It is a major complication of many advanced cancers and seriously affects the quality of life and survival of cancer patients. However, the specific molecules that mediate cancer-related cachexia remain elusive, and the fundamental cellular and molecular mechanisms associated with muscle atrophy and lipidolysis in cancer patients still need to be investigated. Exosomes, a newly discovered class of small extracellular vesicles that facilitate intercellular communication, have a significant role in the onset and development of various cancers. Studies have shown that exosomes play a role in the onset and progression of cancer-related cachexia by transporting active molecules such as nucleic acids and proteins. This review aimed to provide an overview of exosome developments in cancer-induced skeletal muscle atrophy and adipose tissue degradation. More importantly, exosomes were shown to have potential as diagnostic markers or therapeutic strategies for cachexia and were prospected, providing novel strategies for the diagnosis and treatment of cancer-related cachexia.

**Supplementary Information:**

The online version contains supplementary material available at 10.1186/s12967-024-05201-y.

## Introduction

Cachexia is a complex metabolic syndrome characterized by weight loss, dyshomeostasis of energy metabolism, and systemic inflammation [1, 2]. Etymologically, the word cachexia is derived from the two Greek words *“Kakos”* and *“hexis”* meaning “bad condition” [3]. Cachexia is often associated with malignant and multiple chronic non-malignant diseases such as cancer, chronic obstructive pulmonary disease, chronic heart failure, rheumatoid arthritis, kidney disease, acquired immune deficiency syndrome, and septicemia [2, 4]. It is important to mention that nutritional support therapy is not effective in reversing the progression of cachexia [5]. Cachexia gradually leads to physiological dysfunction, promoting the development of multiple organ dysfunction syndromes. Therefore, preventing cachexia holds significant clinical importance in improving the disease progression and the quality of life of patients.

Cancer-related cachexia occurs in the advanced stages of many malignant tumors and is characterized by persistent wasting of adipose and skeletal muscle [6, 7]. Cancer-related cachexia is always accompanied by lipidolysis, skeletal muscle atrophy, and systemic inflammation, resulting in progressive dysfunction and reduced quality of life. Cancer-related cachexia is a prominent contributor to mortality among cancer patients, with previous research indicating that over 20% of cancer patients’ deaths may be indirectly caused by cachexia resulting from cancer or cancer treatment [3]. Many factors influence the occurrence of cancer-related cachexia. They can be attributed to a variable combination of reduced food intake, multiple abnormalities in carbohydrate, fat, or protein metabolism, and disruptions in hormone metabolism. Moreover, the contribution of cytokine activity, intestinal flora, mood disorders (such as depression), and tumor- or treatment-associated pain cannot be disregarded [8, 9]. Patients with cancer-related cachexia are prone to experiencing fatigue and a severe deterioration of physical health, which not only impairs their life quality but may also weaken the radiotherapy/chemotherapy efficacy, ultimately shortening their survival time [2, 10, 11]. Studies have found that cachexia is an independent risk factor for postoperative complications, adverse reactions, and poor prognosis in patients with cancer and has crucial predictive value for prognosis [12, 13]. The current diagnostic criteria for cancer-related cachexia include malnutrition screening and phenotypical and etiological criteria [14, 15]. Malnutrition screening usually uses the malnutrition screening tool, malnutrition universal screening tool, or Nutrition risk screening 2002. Phenotypical criteria refer to loss or low weight, and patients must meet at least one condition, including weight loss exceeding 5% within six months, body mass index less than 20 kg/m^2^, and decreased muscle mass. Etiological criteria refer to increased acute or chronic systemic inflammation [14, 15]. Due to the limited use of invasive metabolic tests and biopsies for diagnosing cachexia, it is challenging to obtain clinicopathological and biochemical data [10, 16]. Additionally, the precise mechanism remains unclear, resulting in ineffective intervention strategies.

Extracellular vehicles (EVs) are nano- to micro-sized vesicles actively released by cells containing complex cargoes such as nucleic acids, lipids, and proteins [17–19]. EVs can transfer biomolecules between different cells and are crucial in intercellular communication, including communication between different organs [20]. Among the classes of EVs, exosomes are small vesicles originating from the endosomal system with a diameter of less than 200 nm [21, 22]. Exosomes carry many biological macromolecules secreted by donor cells, which regulate the function of recipient cells after being taken up [23]. Exosomes, characterized by their stable bilayer lipid membrane and relatively small diameter, have great significance in facilitating intercellular information transfer. This unique intercellular information transfer system has garnered significant recognition for its pivotal involvement in various physiological and pathological processes [24–26]. Many factors regulate skeletal muscle and adipose tissue decomposition, including cytokines, metabolites, proteins, and nucleic acids. Cancer cell-derived exosomes may play important roles in the communication between cancer tissue and skeletal muscle (or adipose tissue). The nucleic acids, lipids, or proteins in these exosomes may contribute to the biological processes underlying cancer-related cachexia, including regulating lipidolysis and skeletal muscle atrophy [27–29]. Meanwhile, stem cell-derived exosomes have demonstrated therapeutic effects in improving skeletal muscle regeneration [30, 31]. Therefore, this review focuses on the research progress and innovative ideas of exosomes in cancer-related cachexia, hoping to shed light on subsequent research.

## Overview of exosomes

EVs are particles released by the cells with a lipid bilayer and cannot replicate independently. EVs are generally divided into ectosomes and exosomes [19]. Ectosomes are vesicles formed through the direct budding of the plasma membrane, with a diameter ranging from 50 nm to 1 μm. Exosomes originate from endosomes, are released during the exocytosis of multivesicular bodies (MVBs), and have a diameter of 30–150 nm (100 nm on average) [32]. Exosomes contain complex components such as proteins, DNA, and RNA from the cells of their origin, which facilitate far and near intercellular communication, influence multiple physiological processes of cells, and participate in the onset of many diseases.

### The biogenesis of exosomes

The exosome production involves double invading the plasma membrane and forming intracellular vesicles (ILVs)/MVBs [33, 34]. Cells receive extracellular components such as proteins, lipids, and metabolites through endocytosis and plasma membrane invagination. This process results in the creation of early-sorting endosomes (ESEs). The formation of ESEs also involves the intracellular Golgi apparatus and endoplasmic reticulum. ESEs mature into late-sorting endosomes (LSEs) and eventually produce MVBs [23]. The second invagination and encapsulation of the cytoplasmic components of LSEs leads to the production of ILVs, and this process can produce ILVs with different diameters and inclusions depending on the volume of invagination. The formation of MVBs is caused by the inward invagination of the endosomal limiting membrane (double invagination of the plasma membrane), resulting in MVBs containing multiple ILVs (future exosomes). MVBs can fuse with lysosomes or autophagosomes for degradation or with plasma membranes to release ILVs as exosomes [23].

### Mechanisms of exosomes involved in intercellular communication

Exosomes can alter the function and phenotype of other cells by carrying a range of nucleic acids, proteins, and metabolites [35]. Exosomes rely on internalization and intracellular transport to traverse the intercellular barrier and transfer vesicle contents. The uptake patterns of exosomes include clathrin-dependent endocytosis, caveolin protein-mediated uptake, lipid raft-mediated endocytosis, macrophagocytosis, and macropinocytosis [36, 37]. Their specificity towards certain cell types enhances the functional complexity of exosomes in intercellular communication. For example, endothelial cells and cardiomyocytes can take up the stem cell-derived exosomes through macropinocytosis [38]. Exosomes secreted by the mesenchymal stem cells can be transported to the recipient cell lysosomes through clathrin-mediated endocytosis [39]. The precise relationship between distinct patterns of exosome uptake by receptor cells and the subsequent localization, degradation, and functional outcomes of exosome components remains uncertain. The fusion of the internalized exosome with the lysosome/endosome leads to the release of cargo into the cytoplasm and the transmission of cellular information from the donor cell to the recipient cell [23]. Interestingly, exosomes that enter the cell can also fuse with ESEs and be rereleased outside the cell as exosomes.

## Cancer cell-derived exosomes promote the formation of cancer-related cachexia

**Fig. 1:**
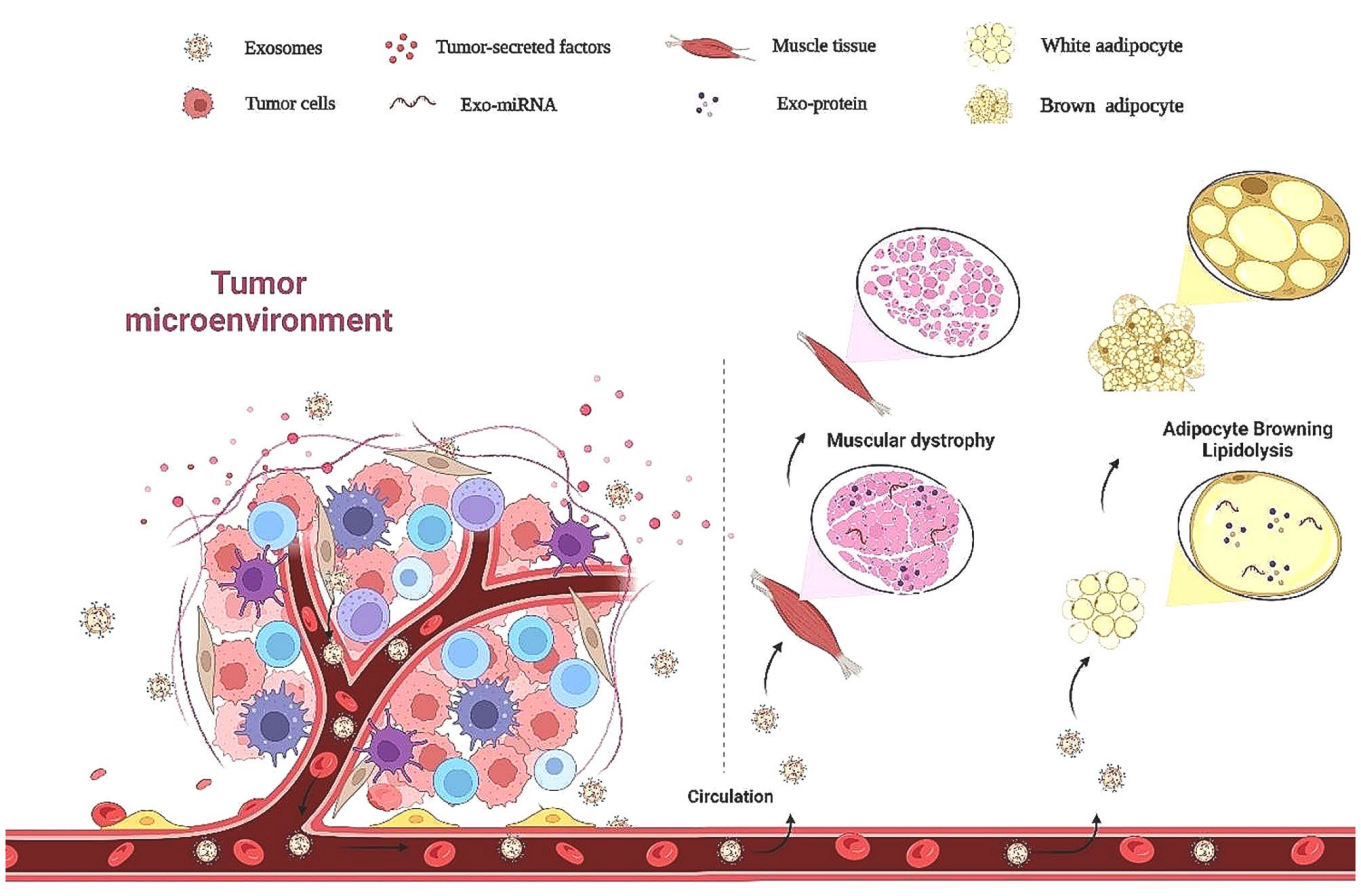
The role of exosomes secreted by tumor cells in cancer-related cachexia. The decomposition of skeletal muscle and adipose tissue induced by malignant tumors was regulated by many factors, including exosomes. Compared to normal cells, malignant tumor cells secrete more exosomes, and tumor cell-derived exosomes could affect other organs, such as adipose tissue and muscle, triggering lipidolysis and muscle atrophy, leading to cancer-related cachexia.

Studies have shown that malignant tumor cells secreted more exosomes than normal cells [40]. This difference suggests that exosomes derived from cancer cells could be emerging mediators of tumorigenesis and cancer-related cachexia (Fig. 1). Exosomes derived from cancer cells not only regulate the growth and metastasis of tumor cells [24, 41] but also affect the physiological activities of neighboring and distant cells, mediate the catabolism process of distal tissues, and increase the destruction of adipose and muscle tissues [23, 42, 43] (Table 1). Regulating the biogenesis of tumor-derived exosomes plays a crucial role in cancer-related cachexia by influencing the efficacy of tumor cells in promoting muscle atrophy and lipolysis [44]. Cancer-derived exosomes can either directly increase muscle loss through inflammatory processes and catabolic factors secretion or promote adipose tissue consumption through increased browning of the white adipose tissue (WAT), abnormal lipolysis, and inhibition of adipogenesis [45]. Existing research on cancer-related cachexia primarily focused on animal models due to the limitations of obtaining clinical samples. Many animal studies have verified the regulatory effect of cancer-derived exosomes, suggesting that they play key roles in adipose tissue degradation and muscle atrophy induced by malignant tumors [46–49].

**Table 1:** Cancer cells promote cachexia through secreting exosomes

Symptoms	Tumor types	Target cell type	Key molecular	Mechanism	Phenotype	References
Lipidolysis	Breast Cancer	Adipocytes	miR-204-5p	VHL/HIF1α/leptin pathway	WAT Browning	[65]
	Colorectal Cancer	Adipocytes	miR-146b-5p	HOXC10/ PRDM16 pathway	Beige/Brown Adipocytes Differentiation	[66]
	Lung Carcinoma	Adipocytes	miR-425-3p	cAMP/PKA signaling	Adipocyte lipolysis and WAT Browning	[71]
	Gastric Cancer	Adipocytes	ciRS-133	PRDM16 pathway	Beige/Brown Adipocytes Differentiation, Glucose Consumption	[73]
	Lewis Lung Carcinoma	Adipocytes	PTHR	PTHrP/PKA/HSL pathway	WAT Browning	[75]
	Lewis Lung Carcinoma	Adipocytes	EIF5A	GPBAR1/cAMP/PKA/CREB pathway	Adipocytes lipidation and wasting	[77]
	Pancreatic Cancer	Adipocytes	Adrenomedullin	ADMR/ERK1/2/p38/HSL pathway	WAT Browning	[79]
	Lewis Lung Carcinoma	Adipocytes	IL-6	STAT3 pathway		[80]
	Lewis Lung Carcinoma	Adipocytes	IL-8	NF-κB pathway	Adipocytes wasting	[81]
	Colon Cancer	Adipocytes	IL-8	NF-κB pathway	Adipocytes wasting	[81]
Reduced adipogenesis	Chronic Myeloid Leukemia	Adipose Tissue-Derived Mesenchymal Stem Cells	miR-92a-3p	C/EBPα pathway	Anti-Adipogenic	[83]
	Lung Carcinoma	Preadipocytes	miR-425-3p	GATA2, IGFBP4, MMP15 and C/EBPα	Inhibit preadipocytes proliferation and differentiation	[71]
	Breast Cancer	Preadipocytes	miR-155	UBQLN1/PPARγ pathway	Inhibit the adipogenesis, WAT Browning	[85]
	Gastric cancer	Adipose mesenchymal stem cells	miR-155	C/EPBβ pathway	Inhibit the adipogenesis and promote brown adipose differentiation	[86]
	Lung Cancer	Adipose Tissue-Derived Mesenchymal Stem Cells	TGF-β	SMAD2/ SMAD4 PPARγ pathway	Anti-Adipogenic	[87]
Muscle atrophy	Osteosarcoma	Muscle-Derived Stem Cells		Notch pathway	Inhibit myogenic potential	[90]
	Pancreatic Cancer	Skeletal Muscle Cells		P38/CEBPβ/UBR2/Atrogin1 pathway	Muscular duct atrophy	[91]
	Lung Cancer	Skeletal Muscle Cells	miR-21	TLR7/ JNK pathway	Apoptosis	[92]
	Pancreatic Cancer	Skeletal Muscle Cells	miR-21	TLR7/ JNK pathway	Apoptosis	[92]
	Pancreatic Cancer	Skeletal Muscle Cells	miR-125b-5p, miR-540-3p, miR-450b-3p, miR-666-3p	PI3K/Akt/FoxO1 pathway	Insulin resistance	[14]
	Colon Cancer	Skeletal Muscle Cells	miR-125b-1-3p, miR-195a-5p	Bcl-2/caspase3 pathway	Apoptosis	[28]
	Oral Squamous Cell Carcinoma	Skeletal Muscle Cells	miR-181a-3p	Endoplasmic reticulum stress pathway	Apoptosis	[94]
	Breast Cancer	Skeletal Muscle Cells	miR-155	PPARγ/ERK1/2 pathway	Cell Catabolism	[95, 96]
	Colon Cancer	C2C12 cells	miR-183-5p	Smad3 pathway	Protein degradation	[97]
	Lewis Lung Cancer	C2C12 cells	IL-6	STAT3 pathway		[80]
	Pancreatic Cancer	C2C12 cells	Hsp70, Hsp90	p38 MAPK pathway		[91]
	Colon Cancer	C2C12 cells	GDF-15	Bcl-2/caspase3 pathway	Apoptosis	[86]
	Lung Cancer	Mesenchymal Stem Cells	Hsp70	TLR2/NF-κB pathway	Pro-Inflammatory	[98]
	Lung Cancer	Skeletal Muscle Cells	Hsp70, Hsp90	TLR4/p38/C/EBPβpathway	Cell Catabolism	[99]
	Lung Cancer	Immune Cells	Hsp70, Hsp90	TLR4/IL-6 pathway	Pro-Inflammatory	[99]
	Esophageal Squamous Cell Carcinoma	Skeletal Muscle Cell	P4HB	Bcl-2/caspase3 pathway	Apoptosis	[100]
	Glioblastoma	C2C12 cells	PAI-1	STAT3 and mTOR pathway	Inhibit muscle protein synthesis	[107]

### Induction of lipidolysis by cancer-derived exosomes

**Fig. 2:**
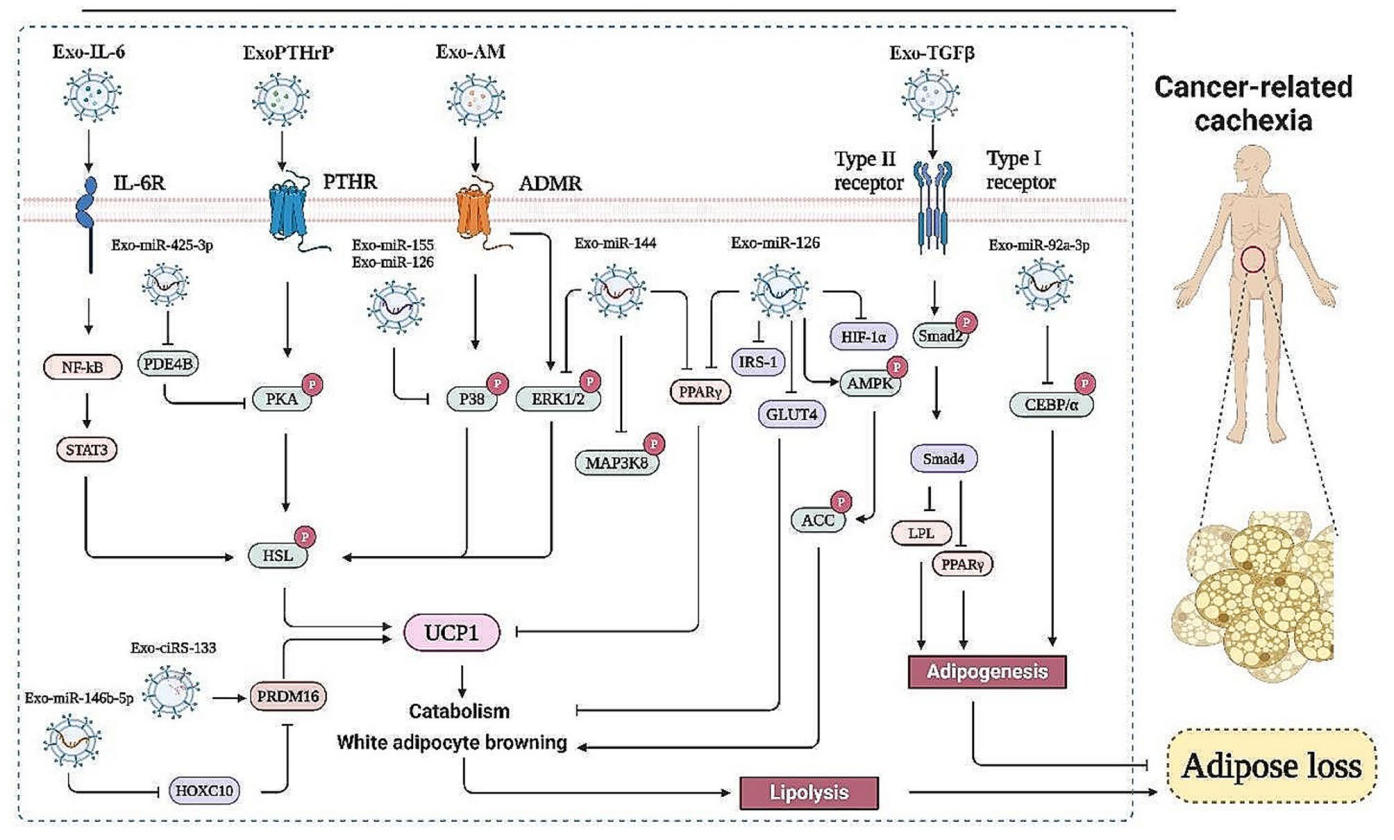
Cancer-derived exosomal non-coding RNAs and proteins promote cancer-related cachexia. Non-coding RNAs and proteins in tumor cell-derived exosomes promote lipid loss by regulating intracellular lipid synthesis and catabolism and participate in cancer-related cachexia

Adipose tissue is important in glucose homeostasis, lipid metabolism, thermogenesis, and insulin sensitivity. One of the hallmarks of cancer-related cachexia is lipidolysis of adipose tissue. Cancer-derived exosomes can promote the decomposition of adipose tissue. GW4869, a neutral sphingomyelinase inhibitor known to block the generation and release of exosomes, could alleviate the lipidolysis of adipose tissue, thereby reducing weight loss caused by cancer-derived cachexia [50]. These findings indicate that nucleic acids or proteins in exosomes have vital functions in the lipidolysis process. Therefore, it is essential to regulate lipolysis in cancer-related cachexia treatment. The detailed information is shown in Fig. 2.

#### Cancer-derived exosomes induce lipidolysis through non-coding RNA

It has been demonstrated that most RNA transcripts do not encode proteins, and genes that encode proteins account for less than 2% of the entire genome [51]. Non-coding RNAs (ncRNAs) are involved in gene regulation at different levels in cellular physiology and shape cellular functions [52, 53]. There are many types of ncRNAs, including microRNAs (miRNAs), long ncRNAs (lncRNAs), circular RNAs (circRNAs), small nucleolar RNAs, piwi-interacting RNAs, and yRNAs [54]. miRNAs are a class of short RNAs derived from long stem-loop structures. In addition to binding and inhibiting messenger RNA (mRNA), recent studies have found that miRNAs can also inhibit proteins, encode short peptides, hinder mitochondrial transcription, activate toll-like receptors, and inhibit nuclear ncRNAs, making miRNAs complex and multifunctional molecules [55, 56]. lncRNAs are ncRNA molecules containing more than 200 nucleotides. lncRNAs have both *cis*-type (performed near the transcription site) and *trans*-type (performed far from the transcription site) functions [57]. The typical *cis*-type functions are associated with chromosomal looping, chromatin modification, and DNA transcription, while *trans*-type functions include binding to proteins and altering their function, attaching to mRNAs and altering their stability, and interacting with other ncRNAs [58]. circRNAs are covalently bonded, closed, uninterrupted loops of nucleotides, making them more stable than other ncRNAs. circRNAs are also called super sponges, as they can bind to dozens of miRNAs and inhibit their function [59]. In addition, circRNAs can also encode micropeptides and binding proteins and regulate their function. Currently, the function of circRNAs has only been partially characterized [56]. While ncRNAs predominantly operate intracellularly, they can also be present in the bloodstream and transported by extracellular vesicles. These systemic circulating ncRNAs originate from specific cell types and can be directly transferred to multiple cells, thereby influencing the functionality of the recipient cells. ncRNAs, especially miRNAs, which are highly expressed in cancer cells, are also enriched in cancer cell-derived exosomes, and can induce lipidolysis.

Adipose tissues are divided into WAT and brown adipose tissue (BAT) and usually perform opposite physiological functions [60]. The primary function of WAT is to store surplus energy in the form of triglycerides and aid in energy accumulation, while BAT acts mainly to produce heat to combat obesity and cold and contribute to energy dissipation [61]. The induction of brown adipocytes within depots of WAT is referred to as WAT browning. WAT browning and lipolysis are complex and involve metabolic processes in which peroxisome proliferator-activated receptor gamma coactivator 1-alpha (PGC1α) and uncoupling protein 1 (UCP1) are markers of lipolysis activation [62]. UPC1 is primarily found in brown or beige adipocytes, facilitating mitochondrial respiration and electrochemical energy conversion into heat to enhance lipolysis, resulting in the depletion of adipose tissue [63]. The rate of fat loss is higher than lean tissue loss in cancer-related cachexia [64]. Also, miRNAs in cancer-derived exosomes affect fat metabolism and regulate WAT browning during cachexia [65, 66].

By targeting the *von Hippel-Lindau* (*VHL*) gene, the exosome miR-204-5p secreted by the breast cancer cells increased the expression of hypoxia-inducing factor 1 α (HIF1α) in WAT, activating downstream leptin signaling pathway, and thereby enhancing lipolysis in WAT. On the contrary, exogenous *VHL* expression blocked the effect of exosome miR-204-5p on WAT Browning [65]. Based on these findings, exosomal miRNAs may facilitate interactions between cancer cells and adipocytes and promote cancer-related cachexia by inducing beige/brown differentiation and enhancing catabolism in recipient adipocytes.

Homeodomain-containing gene C10 (*HOXC10*) was initially identified to regulate cell proliferation and differentiation [67]. Subsequently, *HOXC10* was expressed explicitly in the abdomen’s subcutaneous region and correlated with body fat mass [68]. Upregulation of *HOXC10* levels in cold-exposed mice inhibited the levels of PR domain-containing 16 (PRDM16), a transcription factor regulating the brown adipocyte formation in the subcutaneous WAT, which interfered with the WAT browning to promote lipid accumulation, indicating that *HOXC10* could act as an important negative regulator of WAT browning [69]. Exosomes enriched in miR-146b-5p were released by colorectal cancer cells, which also caused an acceleration of lipolysis. Furthermore, overexpression of miR-146b-5p directly inhibited its target gene, *HOXC10*, causing an increase in WAT browning, a decrease in oxygen consumption, and the induction of fat tissue loss [66].

Research has shown that high levels of circulating exosome miR-425-3p were positively associated with poor progression-free survival in patients with non-small cell lung cancer (NSCLC), and the levels of miR-425-3p could be used to predict the clinical sensitivity of patients with NSCLC to platinum chemotherapy [70]. UCP1 is highly expressed in BAT and is considered to be a marker of lipidolysis activation [62]. Both exogenous miR-425-3p mimics and lung cancer cell-derived exosomes rich in miR-425-3p could stimulate mature adipocytes to release glycerol and increase UCP1 content [71]. This indicates lung cancer cell-derived exosomes could promote adipocyte lipolysis and WAT browning through miR-425-3p. PDE4B is highly expressed in mature adipocytes and is involved in the adipocyte regulation. *PDE4B* gene was identified as the direct target gene of miR-425-3p by bioinformatics analysis and luciferase reporter gene assay. Exosomal miR-425-3p also upregulated intracellular cAMP concentration, downregulated PDE4B, and regulated lipophagy, lipolysis, and WAT browning by activating the cAMP/PKA pathway [71].

circRNAs are another novel family of ncRNAs. circRNAs delivered by exosomes have also been reported to be involved in regulating WAT browning [72]. ciRS-133, found in exosomes from gastric cancer patients and animal models, can be introduced into preadipocytes to stimulate their differentiation into brown-like cells. This is achieved by activating PRDM16, which regulates the metabolic activity of adipocytes and worsens tumor cachexia [73]. Furthermore, in vitro experiments found that tumor-secreted ciRS-133 accelerated oxygen and glucose consumption by BAT, and silencing ciRS-133 could reduce gastric cancer-induced cachexia in mice [73].

These findings demonstrate that tumor cell-derived exosomes can contribute significantly to the pathogenesis of cancer cachexia by promoting WAT browning and adipose tissue degradation via enriched nucleic acid molecules like circRNAs and miRNAs. However, reducing the effects of these exosomes may be required to alleviate cancer-related cachexia.

#### Cancer-derived exosomes induce lipidolysis through enriched proteins

Proteins in exosomes can also affect adipose tissue metabolism and participate in cancer-related cachexia. For instance, the parathyroid hormone receptor (PTHR) is highly expressed in adipose, kidney, bone, and muscle tissues. The parathyroid hormone-related protein (PTHrP) can stimulate the expression of thermogenic genes by binding to PTHR. PTHrP also could facilitate lipolysis by phosphorylating hormone-sensitive lipase (HSL) through protein kinase A (PKA) [74]. Lewis lung carcinoma (LLC) cell-derived exosomes have been reported to stimulate lipolysis in cultured 3T3-L1 adipocytes and WAT of mice with LLC [75]. LLC-derived exosomes could directly fuse with 3T3-L1 adipocytes and transfer PTHrP into 3T3-L1 cells, activating the PKA signaling pathway. Conversely, while blocking PTHrP, reducing PTHR expression or suppressing LLC-exosomes release could alleviate WAT browning and lipolysis [75], indicating that exosomal PTHrP mediated LLC-induced lipolysis.

Eukaryotic translation initiation factor 5 A (EIF5A) is involved in the synthesis and degradation of DNA, RNA, and proteins, and its high expression was associated with poor prognosis in patients with lung cancer [76]. A study revealed that the LLC cell-derived exosomes exhibited a high level of EIF5A, and an upregulation of EIF5A expression was linked to a reduction in the overall survival of patients with lung cancer [77, 78]. The LLC cell-derived exosomal EIF5A was involved in adipocyte lipidation and wasting [77, 78]. Further studies found that EIF5A is directly bound to the GPBAR1 mRNA of adipocytes and regulates its translation. This, in turn, activated the cAMP-PKA-CREB signaling pathway, promoting the transcription of PGC1α and UCP1, thereby inducing lipidation and lipolysis of adipocytes [77].

Both pancreatic cancer cell-derived exosomes, and exosomal adrenomedullin (AM) promoted lipolysis in the human and murine adipocytes. AM could interact with the adrenomedullin receptor (ADMR) on the adipocytes, activate ERK1/2 and p38 pathways, and promote lipolysis by phosphorylating HSL, which could be inhibited by blocking ADMR [79]. Interleukin (IL)-6 in LLC-exosomes induced the lipidolysis of 3T3-L1 adipocytes by activating the signal transducer and activator of transcription 3 (STAT3) and promoting the cachexia process and the neutralization of extracellular IL-6 prevented the lipolysis effects of LLC-exosomes [80]. LLC cell- and C26 cell-derived exosomes induced adipocyte lipolysis and wasting in the diseased mice [81]. Specifically, the effects were primarily triggered by IL-8 in the exosomes. Circulating exosomes from tumor-bearing mice showed a significant increase in IL-8 levels, while PGC1α and UCP1 were significantly upregulated in the adipose tissue of LLC- and C26-bearing mice. However, the lipolysis of adipocytes induced by the LLC cell- or C26 cell-derived exosomes was alleviated by specific IL-8 neutralizing antibodies that blocked IL-8/C-X-C chemokine receptor type 2 (CXCR2) [81]. Furthermore, activating the NF-κB pathway in adipocytes could serve as a key mechanism of lipolysis, and both CXCR2 and NF-κB inhibitors reduced the lipolysis of adipocytes induced by LLC or C26 cells [81]. This suggests that LLC and C26 cells induced adipocyte depletion by activating the CXCR2 receptor on the adipocyte membrane and subsequently activating the downstream NF-κB signaling pathway through the exosome IL-8. These findings indicate that in addition to many ncRNAs carried in cancer-related cachexia, a variety of proteins rich in exosomes likely regulate the occurrence and development of cancer-related cachexia.

### Reduced adipogenesis by cancer-derived exosomes

Cancer-related cachexia is caused by increased lipolysis in mature adipocytes and differentiated preadipocytes and impairment of adipogenesis (Fig. 2). Adipogenesis is a highly regulated process, and the coordinated activation of multiple transcription factors controls the expression of adipogenesis-specific genes. Cancer cell-derived exosomes could inhibit adipogenesis and reduce total fat mass.

#### Cancer-derived exosomes reduce adipogenesis through ncRNAs

Adipogenesis is a complex regulated process, and the adipose tissue-derived mesenchymal stem cells (AD-MSCs) can differentiate into adipose precursor cells and eventually into lipid assimilating cells [82]. Chronic myeloid leukemia (CML)-derived exosomes caused a significant decrease in body weight and body fat rate [83]. Further research found that CML-derived exosomes could be absorbed by the adipose tissue, thereby inhibiting the adipogenic ability of AD-MSCs. miR-92a-3p, enriched in CML cells and exosomes, may play valued roles in this process [83]. After internalization by the adipose tissue, the exosomal miR-92a-3p inhibited the adipogenesis of AD-MSCs by reducing the expression of CCAAT/enhancer binding protein-alpha (C/EBPα) at the post-transcriptional level, and transfection of miR-92a-3p inhibitor blocked the anti-adipogenic effect of CML-derived exosomes [83].

miR-425-3p levels were significantly increased in cachexia-inducing tumor A549, H1299, and AGS cells compared to the non-tumorigenic NL20 and GES-1 cells, and lung cancer cell-derived exosomal miR-425-3p inhibited the differentiation and proliferation of human preadipocyte-visceral (HPA-v) cells [71]. TargetScan analysis and luciferase reporter assay showed that the genes related to the proliferation or differentiation, including *GATA2*, *IGFBP4*, *MMP15*, and *C/EBPα*, were all direct target genes of mir-425-3p, and the lung cancer cell-derived exosomes significantly downregulated the expression of GATA2, IGFBP4, MMP15, and C/EBPα in HPA-v cells [71].

Peroxisome proliferator-activated receptors (PPARs) were expressed in many tissues, such as adipocytes. PPARγ is mainly involved in regulating lipid biosynthesis, energy balance, and adipogenesis [84]. The breast cancer cell-derived exosome miR-155 inhibited the adipogenesis of preadipocytes and promoted WAT browning [85]. Treatment with exosomes could reduce the levels of PPARγ, AdipoQ, and leptin in adipose cells, increase the expression of phosphate hormone-sensitive lipase (P-HSL), UCP1, and adipose triglyceride lipase (ATGL), and promote the release of glycerol. Exosome miR-155 specifically targeted UBQLN1 in adipocytes, and the increased expression of UBQLN1 reversed the fat loss in adipocytes and the brown fat switch caused by the breast cancer-derived exosome [85]. Gastric cancer cell-derived exosomes inhibited lipogenesis in adipose mesenchymal stem cells (A-MSCs), as evidenced by decreased intracellular lipid droplets, and brown fat differentiation with high mitochondrial density was also observed [86]. This phenomenon was mainly related to the abundance of miR-155 in exosomes. Excessive expression of miR-155 in gastric cancer cell-derived exosomes caused cachexia in tumor-bearing mice while reducing exosome miR-155 concentration alleviated cachexia [86]. Mechanistically, exosomal miR-155 secreted by gastric cancer cells inhibited adipogenesis and promoted the differentiation of brown fat by targeting CCAAT/enhancer binding protein-beta (C/EPB-β), accompanied by a downregulation of C/EPB-α and PPARγ expression and upregulation of UCP1 expression [86].

These results provide novel insights into the underlying mechanisms of cancer-induced cachexia, indicating exosomal miRNAs could be the main mediators for cancer-related cachexia. Targeting these specific miRNAs in therapies could offer innovative approaches for clinical intervention.

#### Cancer-derived exosomes reduce adipogenesis through enriched proteins

In addition to ncRNAs, the function of AD-MSCs is also affected by other signaling pathways. AD-MSCs can differentiate into different kinds of cells, including adipocytes. Lung cancer A549 cell-derived exosomes could be internalized by AD-MSCs, resulting in a significantly reduced expression of adipogenic-specific genes. This inhibition of the adipogenic effect of AD-MSCs may be attributed to the TGFβ signaling pathway [87]. TGFβ was found to be abundant in A549 cell-derived exosomes. Following internalization of the exosomes, the TGFβ within the exosomes entered AD-MSCs, inhibiting adipogenic-specific lipoprotein lipase (LPL) and PPARγ, thereby impeding adipogenesis. Additionally, it led to the nuclear localization of SMAD4 [87]. Therefore, these studies shed light on understanding the complexities of cachexia signaling events, and cancer cell-derived exosomes and their enriched macromolecules will be effective potential therapeutic targets for cancer-related cachexia.

### Induction of muscle atrophy by cancer-derived exosomes

**Fig. 3:**
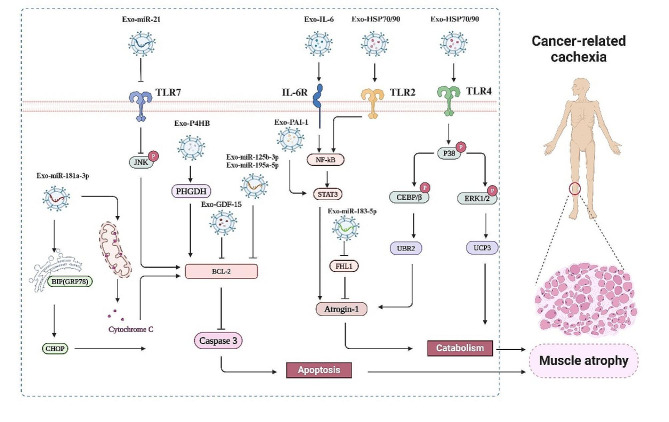
Non-coding RNAs and proteins in tumor cell-derived exosomes promote muscle atrophy and participate in cancer-related cachexia

Skeletal muscles are highly plastic tissues that adapt to various physiological and pathological stimuli, producing structural and metabolic changes [88]. Skeletal muscles function as the body’s primary protein reservoir for energy generation while maintaining metabolic equilibrium in other organs. In pathological states such as cancer cachexia, the presence of malignant tumor metabolites can disturb the equilibrium of muscle homeostasis, leading to the impairment of tissue function and metabolism [89]. Cancer-induced skeletal muscle atrophy is mediated by the interaction of various tumor factors, including exosomes, with skeletal muscle and muscle stem cells (Fig. 3). For instance, coculturing muscle-derived stem cells (MDSCs) with osteosarcoma cells or treating MDSCs with osteosarcoma cell-derived exosomes activated the Notch signaling pathway, decreasing the myogenic potential of MDSCs [90]. Exosomes from pancreatic cancer cells reduced myosin heavy chain (MHC) expression by activating the P38/CEBPβ/UBR2/Atrogin1 signaling pathway in skeletal muscle cells, leading to muscular duct atrophy and muscle weight loss in the cachexia model of mice with in situ pancreatic cancer [91].

#### Cancer-derived exosomes induce muscle atrophy through ncRNAs

Toll-like receptors (TLRs) are necessary for extracellular vesicles to facilitate cellular inflammatory conditions, and exosome miRNAs can control the release of pro-inflammatory cytokines through TLR signaling on the cell membrane. It was found that miR-21 in lung and pancreatic cancer cell-derived exosomes could bind to TLR8 on human muscle cells or TLR7 (homologous to TLR8 in humans) on mouse muscle cells to activate the Jun N-terminal kinase (JNK) pathway and induce apoptosis of muscle cells, thereby causing muscle atrophy [92, 93]. In addition to inflammatory pathways, exosomal miRNAs act directly on apoptosis-related molecules in muscle cells. Colon cancer cells C26-derived exosomes induced skeletal muscle atrophy, decreased myotube formation in vitro, and reduced tibialis anterior muscle weight and strength in vivo [28]. Further results clarified that miR-125b-1-3p and miR-195a-5p might be active biomolecules in C26-derived exosomes. The potential mechanism by which C26-derived exosomal miRNAs (miR-125b-1-3p and miR-195a-5p) induce muscle atrophy in colon cancer-related cachexia may involve BCL-2-mediated myotube apoptosis [28]. Exosomes secreted by oral squamous cell carcinoma cells induced muscle cell apoptosis and muscle atrophy by activating the endoplasmic reticulum stress pathway, and exosomal miR-181a-3p might be a key modulator of this process [94]. These findings indicate that skeletal muscle cell apoptosis may be an important mechanism by which cancer-derived exosomes induce muscle atrophy.

In addition to causing skeletal muscle loss by inducing apoptosis, cancer-derived exosomes can also cause skeletal muscle atrophy by promoting muscle cell catabolism. For instance, breast cancer cell-derived exosomes could induce muscle catabolism of mature muscle cells, leading to loss of myosin heavy chain 1 (MYH1) and myotube atrophy. This mechanism was related to the miR155 in exosomes, and cancer cell-secreted miR-155 significantly downregulated phosphorylated PPARγ (p-PPARγ) and phosphorylated extracellular regulated protein kinases1/2 (p-ERK1/2) and remodeled metabolic process in skeletal muscle cells, leading to breast cancer-associated cachexia [95, 96]. Pancreatic cancer cell-derived exosomes inhibited glucose intake and promoted lipid deposition, causing skeletal muscle atrophy by inducing insulin resistance [27], which was partly controlled by the PI3K/Akt/FoxO1 signaling pathway, and exosomal miRNAs such as miR-125b-5p, miR-540-3p, miR-450b-3p, and miR-666-3p may potentially contribute to pancreatic cancer-induced cachexia [17]. These findings suggest that cancer-derived exosomes induce muscle atrophy by regulating the “metabolic reprogramming” of skeletal muscle cells, paving the way for discovering the potential targets to correct metabolic disorders in skeletal muscle cells and improving cancer-related cachexia treatment/diagnosis strategies.

An enrichment of miR-183-5p was observed in C26 cells and C26 exosomes. Furthermore, the induction of muscle duct atrophy in C2C12 cells by miR-183-5p mimics was accompanied by increased expression of Atrogin-1, MuRF-1, myostatin, HIF-1α, p-SMAD3, and p-STAT3, along with decreased mitochondrial respiration [97]. Four and a half LIM domain protein 1 (FHL1) plays a role in muscle growth, differentiation, and myomas formation, and it is also recognized as a regulator of skeletal muscle mass. Additionally, *FHL1* gene has been identified as a direct target of miR-183-5p. By reducing the expression of Atrogin-1, MuRF-1, and myostatin, inhibiting the activation of the Smad3 pathway, and preserving the mitochondrial respiration, Carnosol has a protective effect on mir-183-5p-induced muscular tube atrophy of C2C12 cells [97].

#### Cancer-derived exosomes induce muscle atrophy through enriched proteins

In addition to ncRNAs in the exosomes, other biomacromolecules like proteins induce skeletal muscle atrophy. IL-6 in exosomes of LLC cells could activate STAT3, which induces C2C12 myotube atrophy [80]. Pancreatic cancer cells increased the levels of heat shock protein 70 (Hsp70) and heat shock protein 90 (Hsp90) released into the circulation through exosomes, leading to muscle atrophy [91]. Hsp70 in the exosomes of A549 lung cancer cells could bind to the TLR2 receptor and activate the NF-κB pathway, resulting in an increased secretion of inflammatory factors, including macrophage chemotactic protein-1, IL-6 and IL-8, and skeletal muscle atrophy [98]. Zhang et al. demonstrated that Hsp70/90 in LLC cell-derived exosomes were key factors for muscle loss in LLC-bearing mice [99]. Tumor-released Hsp70/90-expressing exosomes could activate TLR4-p38-C/EBPβ catabolic signaling pathway in muscle cells, resulting in the activation of ubiquitin-proteasome and autophagy-lysosome pathways, eventually leading to the loss of myofibrillar proteins, muscle mass, and muscle strength. Moreover, downregulation of Hsp70/90 expression in cancer cells or neutralizing extracellular Hsp70/90 inhibited cancer-induced muscle catabolism and depletion in cultured myotubes and mice [99]. Furthermore, Hsp70/90-expressing exosomes could also bind and activate TLR2/TLR4 on the surface of immune cells, triggering the innate immune response and systemic inflammation in lung cancer-bearing mice, thereby inducing the synthesis and secretion of pro-inflammatory factors IL-6 and tumor necrosis factor-alpha (TNF-α), eventually stimulating skeletal muscle degradation [99]. Therefore, these findings suggest that cancer cell exosomes not only directly induce muscle atrophy but also impact the body’s overall metabolism, elevate the levels of circulating cytokines, and indirectly contribute to the regulation of cancer cell-derived exosomes in the skeletal muscle microenvironment.

As a protein disulfide isomerase family member, prolyl 4-hydroxylase subunit beta (P4HB) catalyzes thiol-disulfide exchange and assists in disulfide bond formation. P4HB was considered to be an important mediator of muscle atrophy, and P4HB enriched in esophageal squamous cell carcinoma (ESCC)-derived exosomes could induce skeletal muscle cell apoptosis [100]. Further results confirmed that P4HB promoted cell apoptosis by activating the ubiquitin-dependent proteolytic pathway to regulate the phosphoglycerate dehydrogenase/Bcl-2/caspase3 pathway, while P4HB inhibitor CCF642 inhibited muscle cell apoptosis and prevented ESCC-induced weight loss and muscle wasting [100]. This suggests that P4HB in exosomes could be a potential intervention target for cachexia in patients with ESCC.

Growth differentiation factor 15 (GDF-15) is a member of the transforming growth factor beta (TGFβ) superfamily, and GDF-15 has been reported to have important roles in inflammation and apoptosis [101]. Compared to non-cachexic MC38 tumor cells, it was found that GDF-15 concentration in tumor tissue, serum exosomes, and muscle tissue of C26 tumor-bearing mice was higher, and GDF-15 concentration in C26 cells and exosomes was significantly higher than in M38 cells and exosomes [86]. C26 cells could transfer GDF-15 to C2C12 cells through exosomes and cause muscular atrophy in C2C12 cells [86]. The apoptosis of skeletal muscle cells in mice with C26 tumors was elevated, suggesting that GDF-15 can induce apoptosis in muscle tissue through the inhibition of Bcl-2 and activation of the Bax-caspase 3 pathway [86].

In addition to the effects of the tumor itself, treatments for tumors can also induce or accelerate tumor-related cachexia [102, 103]. Studies have shown that ionizing radiation (IR), as a first-line treatment for glioblastoma (GBM), can promote the secretion of exosomes in glioma cells and induce cancer-related cachexia [104]. Plasminogen activator 1(PAI-1) is a serine protease inhibitor implicated in impaired muscle regeneration [105, 106]. IR had induced muscle atrophy in tumor-bearing mice by increasing PAI-1 released by GBM-derived exosomes, leading to cachexia [107]. After reaching skeletal muscle through blood circulation, PAI-1 could directly penetrate the muscle cells and activate MuRF1 and Atrogin1 by increasing STAT3 phosphorylation, aggravating muscle atrophy [107]. In addition, exosome PAI-1 inhibited muscle protein synthesis by inhibiting mTOR signaling, and pharmacological inhibitors inhibited PAI-1 activity, rescuing muscle protein synthesis, and suppressing muscle atrophy [107].

## Exosomes as biomarkers for diagnosis and prognosis of cancer-induced cachexia

Cancer-derived exosomes contain many nucleic acids and proteins, some of which were abnormally changed in patients with cachexia and were frequently associated with the stage of cachexia [65]. Therefore, the contents of exosomes can serve as biomarkers for the diagnosis and prognosis of cancer-related cachexia (Table 2). For instance, Hsp90 and Hsp70 were significantly increased in serum exosomes of LLC-induced cachexia mice, leading to significant skeletal muscle atrophy, and silencing Hsp70/90 could significantly alleviate skeletal muscle atrophy and the onset of cachexia [99]. The findings indicate a strong association between Hsp70 or Hsp90 in LLC-exosomes and lung cancer cachexia. If further clinical research is conducted, these findings could potentially serve as a cachexia-inducing factor for early detection of cachexia.

**Table 2:** Exosomes can be used for the diagnose and treatment of cancer induced cachexia

Function	Tumor Types	Key Molecular	Phenotype	References
Diagnose	Lewis Lung Carcinoma Exosomes	Hsp90, Hsp70		[99]
	Pancreatic Cancer Exosomes	Adrenomedullin		[79]
	Pancreatic Cancer Exosomes	Glypican-1		[109, 110]
Treatment	Mbryonic Stem Cells Exosomes		Macrophage Polarization	[120]
	Human Umbilical Mesenchymal Stem Cells	Circhipk3	Pyroptosis	[121]
	Mesenchymal Stem Cells	miR − 494	Myogenesis, Angiogenesis	[31]
	Engineered exosomes	miR-26a	insulin resistance	[123]
	Physiactisome	Hsp60	Mitochondrial biogenesis	[126]

Pancreatic cancer is one of the cancers with the highest mortality rate in the world. Most patients are already in the advanced stage when they are diagnosed with pancreatic cancer, having a survival time of less than six months. Therefore, improvements in the early detection of pancreatic cancer may help to reduce the high mortality [108]. Weight loss, especially loss of adipose tissues, can be observed several months before the clinical manifestations of pancreatic cancer. The serum levels of exosomal fraction significantly increased in the patients with pancreatic cancer. Furthermore, the pancreatic cancer-derived exosomes activated lipolysis in human subcutaneous adipocytes compared to exosomes from control subjects. Additionally, exosomal AM was a potential mediator in this process [79]. These findings indicate that the early-onset weight loss before the appearance of pancreatic cancer-related symptoms is mainly due to the lipolysis mediated by AM of pancreatic cancer-exosomes, which is essential for early pancreatic cancer diagnosis. The incidence of advanced cachexia in pancreatic cancer was as high as 80%. Serum exosomes of patients with advanced pancreatic cancer were rich in glypican-1 (GPC-1), which has good specificity and sensitivity in detecting pancreatic cancer [109, 110]. Therefore, exosomal GPC-1 might be a potential biomarker for the rapid diagnosis of pancreatic cancer-related cachexia. Although exosomes and exosomal GPC-1 or AM as biomarkers for diagnosing cancers and their induced cachexia still need research in a larger patient group, these discoveries demonstrate the power of exosomes in diagnosing cancers and their complications. It is expected that there will be deeper research on exosomes as diagnostic markers so that exosomes can be used to detect tumors such as pancreatic cancer or lung cancer and their induced cachexia.

Exosomes exhibit greater concentrations and stability in body fluids and have a prolonged half-life, making them advantageous for early cancer detection compared to other markers. Moreover, exosomes can easily reach various body fluids due to their high permeability, providing an important prerequisite for liquid biopsy. The nanohybrid-based integrated electrochemical liquid biopsy (ELB) constructed by Zhang et al. can directly and rapidly detect exosomes in serum [111]. The comparative analysis of serum samples from patients with lung cancer and patients without cancer showed that the detection specificity of the ELB platform was 0.91, and the detection sensitivity was 0.94 [111], which can help in the early detection of lung cancer in the future. Although specific ncRNAs or proteins in exosomes are closely related to the pathological process of cancer-related cachexia, the specificity, and sensitivity of exosomes in diagnosing or predicting cancer-related cachexia have not been systematically studied. More clinical studies may be needed to confirm the role of exosomes in diagnosing or predicting cancer-related cachexia.

## Roles of exosomes in the treatment of cancer-related cachexia

Exosomes not only act as prognostic and diagnostic biomarkers but may also have potential uses in cell-free therapies for cancer-induced cachexia. Now, the commonly used treatment strategies for cancer-induced cachexia include exercise intervention, nutritional management, and pharmacological therapy. Although certain patients may benefit from the above treatments, there are no definitive guidelines for this debilitating condition. Cell-based therapies for muscular dystrophy have been in the experimental phase for several decades. Various types of cells, such as MSCs and pluripotent stem cells, with different characteristics and tissue origins, have been researched, and clinical trials have been conducted. However, immune rejection of transplanted allogeneic cells or expression of exogenous therapeutic genes by autologous cells is a complex problem for stem cell therapy. It is important to minimize the immune response for the success of stem cell therapy [112]. Recent research has shown that the functional advantages of stem cell therapy, to a large extent, were mediated through paracrine functions, and exosomes were the central paracrine functional units [113–115]. Many studies have confirmed that exosomes secreted by stem cells have protective functions similar to stem cells, making them a promising and effective therapy. The benefits of exosomes derived from induced pluripotent stem cells (iPSCs) and MSCs on muscle repair and regeneration have been explored [116–119]. For instance, embryonic stem cell exosomes (ES-Exos) could ameliorate doxorubicin-induced muscle toxicity. ES-Exos treatment remarkably upregulated anti-inflammatory M2 macrophages and decreased pro-inflammatory M1 macrophages in muscle. ES-Exos significantly reduced pyroptosis and inflammasomes, improving muscle function [120]. These results suggest that stem cell-derived exosomes may be potential therapeutics or beneficial carriers of therapeutic agents in improving muscle function.

Stem cell-derived exosomes can also be used as carriers to deliver ncRNAs to repair damaged tissues. Human umbilical MSCs-derived exosomes could enhance ischemic hindlimb repair by delivering circHIPK3 to prevent skeletal muscle pyroptosis and repair muscle injury. circHIPK3 acted as a miR-421 sponge to increase the expression of FOXO3a, and prevent the activation of inflammasomes caspase-1 and NACHT, LRR and PYD domains-containing protein 3 (NLRP3), inhibiting inflammation [121]. This indicates that exosome-based therapy could be an effective therapeutic approach for treating muscular dystrophy. Additionally, MSCs-derived exosomes enhanced myogenesis and angiogenesis, promoting muscle regeneration, and this effect was partly mediated by miRNAs such as miR-494 [31].

The broad application prospects of exosomes as drug delivery systems for treating cancers and cancer-related cachexia can be attributed to their favorable characteristics, including stable structure, low toxicity, and ability to cross physiological barriers. Exosomes can be used as carriers to transport anti-cancer drugs and inhibit drug resistance and metastasis of tumor cells, thereby alleviating muscle atrophy and adipose tissue lipolysis induced by cachexia. For instance, iExosomes (engineered exosomes), which carry short hairpin RNAs or short interfering RNA specific to oncogenic *KRAS*, could inhibit PC cell growth in multiple animal models and significantly increase overall survival [122]. These outcomes provide insights into the potential exosomes as therapeutic vehicles in treating cancers and their induced cachexia. Furthermore, exosomes can also serve as delivery systems for ncRNAs or proteins to promote muscle growth and repair for alleviating cancer-related cachexia-induced muscle atrophy. Several studies have initiated efforts to use exosome-loaded miRNAs to treat muscle atrophy-related diseases. Exo/miR-26a is a genetically modified exosome that could selectively target muscle cells and muscle stem cells. It is rich in miR-26a and embedded with Lamp2b, an exosomal membrane protein gene fused with a muscle-specific surface peptide [123]. . Exo/miR-26a treatment increased miR-26a expression and cross-sectional areas of skeletal muscle, decreased muscle protein degradation, and inhibited muscle atrophy [123]. Exo/miR-26a offers a therapeutic strategy for treating muscle atrophy using exosome delivery of biomolecules or drugs.

Hsp60 is a protein structurally expressed in muscle cells, and its expression level is positively correlated with the mitochondrial content and oxidation capacity of muscle cells [124]. Hsp60 levels were increased with training, and increased Hsp60 levels in the trained mice contributed to the import and folding of mitochondrial proteins, thereby inducing mitochondrial biogenesis [125]. Researchers have designed an Hsp60-rich nanovesicle with a 50–140 nm diameter called Physiactisome. C2C12 myoblasts take up Physiactisome, increasing intracellular PGC-1α, potentially beneficial for muscle atrophy [126]. Hence, Physiactisome exhibits promise as a nanovesicle-derived anti-cachexia drug, capable of replicating the advantageous outcomes of physical activity, thereby enhancing patient survival and quality of life. In addition, Hsp60 can be added to the nanovesicles to deliver other active proteins or drugs.

It is worth noting that the results of clinical studies are still very limited, even though preclinical studies have demonstrated the potential of exosomes in cancer-related cachexia treatment, and many experimental results in animal models have verified the feasibility and effectiveness of exosome therapy. Phase I clinical studies have demonstrated good tolerance towards mesenchymal stromal cell-derived extracellular vesicles in healthy volunteers [127]. Patients with colorectal cancer also have a good tolerance against autologous ascites-derived exosomes [128]. A 12-week prospective, double-blind, randomized, controlled study found that human adipose tissue stem cell-derived exosomes effectively treat acne scars and have a shorter recovery time after treatment [129]. To date, clinicaltrials.gov, a US clinical trial registry, has registered hundreds of clinical trials evaluating the efficacy and safety of exosomes, and much research has been done on stem cell-derived exosomes [130]. With continuous exploration, exosomes may bring much hope for cancer-related cachexia treatment.

## Limitations and prospects of the application of exosomes

Two main obstacles exist to using exosomes as diagnostic markers or therapeutic candidates. The first objective is to streamline and standardize the exosome separation process, enhancing the yield. The second objective is to accurately differentiate exosomes from other extracellular vesicles, exceptionally functional micro-vesicles. Compared to cell and gene therapies, clinical data of internal and external exosomes are limited globally. As a class of potential drugs, exosomes still need more clinical data for individual applications. Nevertheless, further investigation necessitates the support of more sophisticated fundamental technologies, including separation, purification, and detection.

### Limitations and prospects of exosomes separation method

Although cancer cells-derived exosomes have great potential for early diagnosis of cancer and cancer-related cachexia and are also receiving increasing interest, obstacles such as high-efficient isolation methods and ultra-sensitive detection techniques remain [131]. Exosomes are distributed in body fluids with extremely complex contents, which makes high-yield exosome isolation challenging.

Ultrafast centrifugation, subdivided into differential ultrafast centrifugation and density gradient centrifugation, is the gold standard for exosome separation [132]. Differential centrifugation is the most common strategy for exosome separation. However, high protein aggregates and lipoprotein contamination levels in the exosome samples significantly impair their quantitative and functional analysis. Density gradient centrifugation can provide purer exosome samples for downstream applications. However, this technique fails to extract extracellular vesicles, including micro-vesicles that resemble exosomes. In addition, the structure and biological function of exosomes isolated by ultrafast centrifugation may be affected by the prolonged ultra-centrifugal forces, which is detrimental to downstream applications, such as exosome-based functional research and drug development. Ultrafiltration and size exclusion chromatography can isolate exosomes with minimal structural damage and preserve their natural bioactivity [133–135]. However, all these methods rely on cell growth in serum-free media, and lack of serum is a potential stress inducer. Therefore, the content and function of exosomes secreted can be impacted by the cells grown in serum-free media [136].

Polymer-based precipitation is another commonly used exosome separation strategy. It is the basis for several popular commercial exosome separation kits. However, the processing time required by this method is long and requires complex cleaning steps, which will affect the downstream analysis and quantitative results [137]. Furthermore, using particular surface proteins found on exosomes, such as CD9, CD63, or CD81, can be employed to capture these antigens on exosomes using immunomagnetic beads containing modified antibodies, thereby effectively isolating the desired exosomes [138]. Lim et al. applied a novel method using antibody-coupled magnetic nanowires, which increased exosome capture efficiency approximately three-fold compared to magnetic beads [139]. Kabe et al. reported a new technique called ExoCounter that captured exosomes using an optical disc coated with antibodies and quantified the captured exosomes [140]. Cross-sectional cohort analysis of serum samples using the ExoCounter system showed a significant rise in receptor tyrosine-protein kinase erbB-2 (HER2)-positive exosomes in patients with breast or ovarian cancer compared to healthy individuals and patients without cancer [140]. A novel exosome separation strategy based on the specific interaction between the immobilized peptide ligands and the highly abundant phosphatidylserine portion of the exosome surface makes it possible to isolate the exosomes with high yield in a short span [141]. The latest microfluidic technology is a multifunctional tool for exosome preparation that facilitates real-time analysis of exosomes. This technology allows for rapid isolation of exosomes from trace amounts of body fluids and enables real-time exosome characterization for in situ diagnosis. These innovative technologies may dominate future advancements in exosome separation [142, 143]. However, despite advancements in separation and purification techniques, the reduction of preparation costs and the implementation of large-scale production continue to be significant obstacles.

### Limitations and prospects of exosomes as therapeutic candidates for cancer-induced cachexia

Exosomes, naturally occurring nanoscale transport vesicles, possess several advantageous properties not found in synthetic vesicles or cells. These include targeted modification, high permeability, low immunogenicity, and high tolerance. Therefore, its potential application in therapy has attracted increasing attention. Exosomes applying surface ligand-receptor presentation enable precise targeting, whereas alternative entry modes or interaction mechanisms enable distinct exosomes to regulate their effects on particular target cells. Exosomes are also being used in clinical trials for various indications.

Despite the considerable clinical potential of exosome-delivered drugs for the treatment of cancers and cancer-related cachexia, numerous challenges remain. Exosomes contain various nucleic acids, proteins, and different microenvironments. For instance, hypoxia has a significant impact on the contents of exosomes. Notably, the nature of the contents in exosomes depends entirely on the origin of the cell that releases the exosomes. Therefore, it is important to understand how the contents of exosomes are packaged. While research indicates that exosome biogenesis and secretion are subject to cellular regulation, it remains uncertain whether including contents into exosomes is a selective or random process. Since cancer cells are known for their heterogeneity, the nature of the cargo from each cancer cell is unique. Meanwhile, existing studies have only investigated a few active molecules in exosomes, and the functions and mechanisms of many potentially therapeutic molecules remain to be further studied.

Moreover, exosomes can provide a more feasible way to transform from laboratory to clinic compared to cell-based therapies. However, further continuous optimization is still needed to reduce the inefficient endocytosis and off-target effects and increase stability of exosome-based therapies. The loading capacity of exosomes and methods to enhance their targeting need to be optimized to achieve large-scale clinical applications.

Again, despite its many advantages, using exosomes as a delivery system may need to address some potential issues. Delivering biological molecules or drugs into the correct cellular compartments remains challenging and costly while maintaining their biological potency, integrity, and stability. The current understanding of the pharmacokinetic properties and biological distribution of exosomes is insufficient, and the precise mechanisms by which exosomes interact with target cells and achieve selectivity remain unclear. An in-depth understanding of these processes is required to develop effective therapies targeting exosome communication and iExosome-derived therapeutic vectors.

Finally, to ensure the stability of clinical efficacy, the preparation of exosomes must be standardized, including source selection, isolation, characterization, drug delivery, stability, targeting, and quality control, following good manufacturing practices, which is an important prerequisite for the clinical application of exosomes. Therefore, there is a need to develop guidelines for the manufacture, storage, and management of therapeutically relevant exosomes and follow good manufacturing practice standards for safety and quality.

Future studies can provide greater insights into the biological significance of intercellular transport via exosomes. Systematic studies on the structural and functional biology of exosomes can increase our understanding of their role in cancer-induced cachexia.

## Conclusion

Cachexia is one of the most common complications in patients with malignant tumors. Skeletal muscle atrophy and adipose tissue lipidolysis in patients with malignant tumors affect the quality of life of patients, the efficacy of radiotherapy/ chemotherapy, and shorten the survival of patients, which emphasize the need to develop effective clinical treatment strategies for cancer cachexia. Studies have found that cancer cells secreted more exosomes than normal cells. Exosomes were not only involved in tumorigenesis, drug resistance, and metastasis but also in the development of cancer-related cachexia. Nucleic acids and proteins carried by exosomes can directly or indirectly inhibit lipid synthesis and induce adipose tissue lipidolysis and skeletal muscle atrophy. These make exosomes an important class of regulators, making them an actionable target to improve the onset and progression of cancer cachexia. With the aid of cutting-edge technologies, research on exosomes has achieved some results in the onset, diagnosis, and treatment of cancer-related cachexia.

Despite the clinical translation of exosomes-based therapeutics remains technical challenges, we can still be optimistic about using exosomes as a novel strategy for the diagnosis and treatment of cancer-related cachexia. The increasing sophistication of technological and methodological innovations allows us to overcome many difficult aspects of exosome isolation and standardization and to identify opportunities for therapeutic development. The malleability of exosomes present exciting opportunities for the development of exosomes-based therapeutics for the occurrence, diagnosis, and treatment of cancer-related cachexia.

### Electronic supplementary material

Below is the link to the electronic supplementary material.

Supplementary Material 1

Supplementary Material 2

## Data Availability

All data generated or analysed during this study are included in this published article.
