# Supplementary material for: Exosomes in the pathogenesis and treatment of cancer-related cachexia
Source: J Transl Med. 2024 Apr 30;22:408. doi: 10.1186/s12967-024-05201-y (PMC11062016; doi:10.1186/s12967-024-05201-y)
Supplement: Supplementary file 1 — Supplementary Material 1 [file 12967_2024_5201_MOESM1_ESM.pdf]

Shanghai Keteng Educational Technology Co.,Ltd.
Room 14401, NO.498, Guoshoujing Road, Pudong New Area, Shanghai, China
Contact us: +86 021-50829828 13110130002@alu.fudan.edu.cn

Certificate Number: KT2024030509R

# CERTIFICATE OF ENGLISH EDITING

This document certifies that the manuscript entitled

*"Exosomes in the pathogenesis and treatment of cancer–related cachexia "*

was edited for English language, including grammar, punctuation and spelling by one or more native English-speaking editors of KetengEdit. Neither the research content nor the authors' intentions were altered in any way during the editing process.

Best Regards
Keteng Editing Team
Date Issued
March 24, 2024

**Disclaimer:** The document we edited contains approximately 1000 words, and any changes made may be accepted or rejected by the authors at their discretion following our editing. KetengEdit, however, is not liable for any revisions made to the document after our editing on March 24, 2024.

To verify the final edited version, or if you have any questions or concerns regarding the edited document, please contact us at 13110130002@alu.fudan.edu.cn. For more details regarding our company and current services, please visit: http://www.ketengedit.com
