# Supplementary material for: Exosomes in the pathogenesis and treatment of cancer-related cachexia
Source: J Transl Med. 2024 Apr 30;22:408. doi: 10.1186/s12967-024-05201-y (PMC11062016; doi:10.1186/s12967-024-05201-y)
Supplement: Supplementary file 2 — Supplementary Material 2 [file 12967_2024_5201_MOESM2_ESM.docx]

Dear Editors and Reviewers:

Thank you for your letter and for the reviewers’ comments concerning our manuscript entitled “Exosomes in the pathogenesis and treatment of cancer-related cachexia” (JTRM-D-24-00720). Those comments are all valuable and very helpful for revising and improving our paper, as well as the important guiding significance to our researches. We have studied comments carefully and have made corrections which we hope may meet with approval. Revised portions are marked in red in the revised manuscript. Our point-by-point responses to the reviewers’ comments are provided below. Each original comment is presented, followed by our “**RESPONSE**”.

Response to reviewer #1:

The manuscript appears to address a significant and timely topic in oncology, focusing on the role of exosomes in cancer-related cachexia. Overall, the manuscript seems to contribute to the growing body of research on cancer cachexia and highlights the potential of exosome-based approaches in diagnosing and treating this challenging condition. However, some improvement needed as below:

1. While the manuscript mentions the potential of exosomes as diagnostic markers or carriers for cachexia treatment, it would be beneficial to elaborate on the specific challenges and opportunities associated with translating these findings into clinical practice. For example, discussing the specificity and sensitivity of exosome-based diagnostic assays or the feasibility and efficacy of exosome-based therapeutics in preclinical and clinical settings.

**RESPONSE**: Thanks for reviewer’s suggestions! Although many preclinical studies have confirmed that exosomes are associated with cancer cachexia and may be used as potential diagnostic markers, there are not enough clinical studies to discuss the specificity and sensitivity of exosome-based diagnostic assays. Similarly, stem cell-derived exosomes or engineered exosomes have the potential in the treatment of cachexia, however, there are currently no approved exosome-based therapeutics. In the revised manuscript, we have added specific challenges and current research advances in translating these findings into clinical practice.

2. Limitations and Future Directions: Addressing potential limitations or unanswered questions in the field of exosome research related to cancer cachexia could provide valuable insights for future studies. Discussing areas where further research is needed to elucidate the precise roles of exosomes in cachexia pathogenesis and optimize their diagnostic and therapeutic applications would help guide future research efforts.

**RESPONSE**: Many thanks for the reviewer's suggestions! In the revised manuscript, we have added "Limitations and future directions" to discuss potential limitations or unresolved issues in the current field of exosome research related to cancer cachexia. This part of the content was mentioned in the conclusion before, and we are very sorry that the content before may not be in-depth enough. In the section "Limitations and Future directions" of the revised manuscript, we summarized the progress of existing isolation and detection techniques, and discussed in depth the opportunities and challenges in the current clinical application of exosomes, hoping to provide help for the future application of exosomes in clinical diagnosis and therapy.

Reviewer #2: The review manuscript gives a good overview of the advances of exosomes in cancer-induced skeletal muscle atrophy and adipose tissue degradation.

The authors summarize recent findings in a structured way and the final sections about the use of exosomes as biomarkers and as a therapeutic option for cachexia put forth a very insightful 'take-home message'.

However, there are a few concerns with regard to the grammar and the sentence structures of the manuscript.

1. I would highly recommend the authors to have the manuscript proof-read by a native speaker, since there are many discrepancies with the used tenses. There are also instances of jumping from one concept to another in an unharmonious manner that makes reading and understanding the manuscript quite challenging.
Some examples include: Line 55-58, 133-135, 163-166, 181-184, 283-286, 331-332...

**RESPONSE**: We are very sorry for the inconsistent use of tenses and improper language expression. Thanks for the reviewer's suggestions. The manuscript has been carefully revised by professional native speakers, and we have also carefully revised the contents of the manuscript, especially the parts pointed out by the reviewers.

2. I would also recommend to add a small section about non-coding RNAs, in order to introduce the different types and their potential functions.
**RESPONSE**: Many thanks for the reviewer's suggestions. Before introducing the effects of cancer-derived exosomal non-coding RNAs on lipidolysis, we have added a small section on non-coding RNAs to introduce different types of non-coding RNAs and their potential functions.

3. The authors could also consider using the term small extracellular vesicles instead of exosomes, as has been suggested in the Minimal information for studies of extracellular vesicles 2018 (MISEV2018), and also the upcoming MISEV2023.

**RESPONSE**: Thanks for reviewer’s suggestions! MISEV2023 has just been published (Welsh JA, et al. Minimal information for studies of extracellular vesicles (MISEV2023): From basic to advanced approaches. J Extracell Vesicles. 2024 Feb;13(2): e12404), and we have studied the article carefully. small extracellular vesicles (small EVs) refer to intraluminal vesicles with the diameter generally smaller than 200 nm. exosomes are not recommended for MISEV2023 unless subcellular origin can be demonstrated. In future studies, "exosomes" can only be used when there is sufficient evidence, unless the subcellular origin can be proven and specialized isolation and characterization of small EVs will be more rigorous. However, considering the "exosomes" used in the original literature cited in the manuscript, the revised manuscript still uses the "exosomes". Thank you very much for your advice, we have realized that the use of "exosome" is not rigorous and are working hard to define the term as clearly as possible in future studies.

Reviewer #3: The manuscript titled 'Exosomes in the pathogenesis and treatment of cancer-related cachexia' presents a comprehensive summary of the advances of exosomes in cancer-induced skeletal muscle atrophy and adipose tissue degradation. Overall the topic of the review article is of interest and importance to the field. The authors have attempted to identify the role of exosomes in regulating the progression of cachexia and discuss some strategies to target and modify the exosomes as potential diagnosis and treatment markers for improving cancer-related cachexia. Overall, the manuscript is well written and presents a good summary about the current literature highlighting the role of exosomes in cachexia progression. The concerns or comments associated with the manuscript have been highlighted below:
1. Abstract: The abstract is very weak and general in approach. The lines 12-15 lack a clear transition and seem to be a very general statement to the field. The abstract must be tailored to suit the specific review.

**RESPONSE**: Many thanks for the reviewer's suggestions! We have revised the summary and added transitional sentences in the parts pointed out by reviewers.

2. For the paragraph that ends on line 64 the authors need to discuss in greater details the methods employed to validate the onset of cachexia.

**RESPONSE**: Many thanks for the reviewer's suggestions! In this section, we have added to the research progress in the clinical diagnosis of cancer cachexia.

3. Line 71: The review lacks in defining the what makes exosomes unique compared to other EVs. The criteria that distinguish exosomes from other EVs need to be better parsed out.

**RESPONSE:** Many thanks for the reviewer's suggestions! We have added an "Overview of Exosomes" section, which describes the production process of exosomes and the possible mechanisms of uptake by recipient cells, and compares the differences between exosomes and other extracellular vesicles.

4. For the paragraph starting Line 86: The authors need to detail how exosomes can directly aggravate loss of adipose tissue or muscle.

**RESPONSE:** Many thanks for the reviewer's suggestions! Since we have analyzed in detail the effects of exosomes from different cancer cells on adipose tissue and muscle in the following content, we tried our best to summarize the mechanism of exosomes from cancer cells on adipose tissue and muscle in the part pointed out by the reviewers.

5. Figure 1 is referenced in Line 101 but it does not represent the claims made in the references listed before that line. The figure in fact does not seem very related to the review and a more general depiction of exosomes in cancer. The figure should be modified to better suit the review.

**RESPONSE:** Thanks for the reviewer's suggestions! Figure 1 provides an overall overview of the content of the review. We are very sorry that Figure 1 appeared inappropriately in the manuscript before, and we have made changes to Figure 1.

6. Line 101: Rather than just listing the studies that identify the role of exosomes in adipose tissue degradation and muscle atrophy, detail the mechanism or proposed regulatory pathways. This paragraph seems a restating of existing literature without presenting the underlying perspective.

**RESPONSE:** Thanks for the reviewer's suggestions! Since we have analyzed in detail the effects of exosomes from different cancer cells on adipose tissue and muscle in the following content, in this section, we only briefly outlined the role of exosomes in adipose tissue degradation and muscle atrophy, and summarized the possible mechanisms of action.

7. Figure 2 again does not justify the location where it is referenced. It has many different pathways and extremely complicate. The figures are not helping the reader and need to be streamlined to better align to the paragraph and the underlying messaging.

**RESPONSE:** Thanks for the reviewer's suggestions! We have modified Figures 2 and 3 to make the information more intuitive and readable.

8. Lines 117-122; Lines 133-135; Lines 136-139 are missing references.
**RESPONSE:** Thanks for the reviewer's suggestions! We have carefully checked the full manuscript, and we have added references to the parts pointed out by the reviewers.

9. Paragraph lines 147-158 seem to be placed out of context. Please provide a transition or explanation for the paragraph.

**RESPONSE:** Thanks for the reviewer's suggestions! We have added transitional sentences in the parts pointed out by reviewers.

10. The writing for the section on induction of muscle atrophy by Cancer-derived exosomes needs to be revised to avoid jargon and exaggerated conclusions. The authors seem to overtly extend the original findings from the articles in this section.

**RESPONSE:** We have modified the section about the effect of cancer-derived exosome on muscle atrophy, summarizing the findings of the original literature as objectively as possible. Most thanks for the reviewer's suggestions! Your comments are very important to our future research.

11. The section titled Exosomes as biomarkers for diagnosis and prognosis of cancer induced cachexia should be revisited as it is one of the central features of this review. The authors need to provide a model figure for this section. Conclusions in this section are important and the authors should present them in a succinct manner.

**RESPONSE:** Thanks for the reviewer's suggestions! In this section, we analyzed the progress of exosomes as biomarkers for the diagnosis and prognosis of cancer-induced cachexia. We also tried to use model figure to illustrate this part of content, but due to the lack of close connection between the results of different original literatures, we still used the form of tables to present.

12. The conclusion is very poorly presented with generic statements which does not summarize the findings from the current review manuscript. The conclusion should present the authors perspective based on the current state of research findings related to cancer-related cachexia. The authors should avoid generalized statements related to cachexia and not restate previous conclusions from other published reviews.

**RESPONSE:** Thanks for the reviewer's suggestions! We have revised the conclusion and added "Limitations and future directions" to discuss potential limitations or unresolved issues in the current field of exosome research related to cancer cachexia.

Reviewer #5: In this review by Ru et al, the authors have reviewed the role of exosomes in the pathogenesis and treatment of cancer-related cachexia. The review is nicely summarized. I would recommend the authors to include a section of exosome biogenesis and its molecular cargo before introducing its role in cancer-related cachexia.
**RESPONSE:** Thanks for the reviewer's suggestions! We have added an "Overview of Exosomes" section, which describes the production process of exosomes and the possible mechanisms of uptake by recipient cells, and compares the differences between exosomes and other extracellular vesicles.
